# Sexual Behavior of Perinatally Infected Youth in Northwest Ethiopia: Implication for HIV Prevention Strategy

**DOI:** 10.1155/2018/1573845

**Published:** 2018-11-01

**Authors:** Nurilign Abebe Moges, Habtamu Mellie Bizuayehu

**Affiliations:** Public Health Department, Medicine and Health Sciences College, Debre Markos University, Debre Markos, Ethiopia

## Abstract

**Background:**

The major mode of HIV transmission in many resource-limited settings is via heterosexual intercourse, but the primary risk factor for youth is primarily through perinatal infection. With the maturing of the HIV epidemic, youth who acquired the virus perinatally are now reaching adolescence and becoming young adults. There is a paucity of data on the sexual practices of perinatally infected youth in Ethiopia.

**Methods:**

This a cross-sectional study among 343 HIV positive youths receiving HIV care and treatment in the two hospitals in northwest Ethiopia. A self-administered questionnaire was administered among those who were able to read and write, and the questionnaire was administered by a trained study team member for those who were illiterate. Data were entered using Epi data version 3.5 and analyzed using SPSS. Sexual behaviors of the two groups were compared using bivariate logistic regression and the significant ones were further analyzed using multivariate logistic regression. Statistical significance was declared at 95% confidence interval and P-value less than 0.05.

**Result:**

About (63.3%) were females, and 177 (51.6%) were between 20 and 24 years of age. The modes of HIV acquisition were 133 (35%) through perinatal HIV infection, 120 (35%) through sexual contact, 27 (7.9%) through exposure to HIV infected sharp materials, and 63 (18.4%) unsure how they acquired HIV. More than half 155 (59.3%) had multiple sexual partners, and 50 (63.3%) of their sexual partners were HIV negative. Among those who were sexually active, only 77 (56.2%) use a condom consistently.

**Conclusions:**

More children who acquired HIV from their mothers are joining the youth population. Their sexual behavior is similar to those youth with behaviorally acquired HIV. There is significant risky sexual behavior among both groups. There is great urgency to effectively address the HIV the prevention strategy to break the cycle of “transgenerational” infection.

## 1. Background

Globally, there were a total of 36.7 million (30.8 million-42.9 million) people living with Human Immunodeficiency Virus (HIV) with an estimate of 5000 new infections daily. Among these, 64% are from sub-Saharan Africa, and 37% of new infections are among young people aged 15–24 years [1]. In Ethiopia, new HIV infections are increasing; in 2016 there were 30,000 (19,000–41,000) compared to 2010 when there was an estimate of 23,000 (16,000–31,000) infections, thus reverting to the peak of the epidemic in 2005 when an estimate of 30,000 (23,000–39,000) new infections was reported [1]. In eastern and southern Africa, young women aged 15–24 years accounted for 26% of new HIV infections in 2016 despite making up just 10% of the population [1]. These surveillance data indicate that youths remain at risk of HIV in this region. It has been more than 33 years since HIV was noted for the first time in 1984 in Ethiopia [2]. Most HIV infections in Ethiopia are the result of heterosexual intercourse and currently the percentage of young females age 15–24 who have had their sexual debut before age of 15 has decreased from 16% in 2005 and 11% in 2011 to 9% in 2016 [3]. Currently, there are 710, 000 (570,000–880,000) living with HIV in Ethiopia, and 61% of adults and 35% of children are on treatment [1].

Young people living with HIV are often in discordant sexual relationships, with potential for HIV transmission to their HIV-negative partner. A study in central Uganda revealed that 40% preferred negative partners for marriage and only 31% boy or girl relationships had disclosed their HIV status to their partners. A reported 57% of participants in this study did not consistently use condoms, and 30% had more than one sexual partner in the past six months [4].

There are some studies on the sexual risks behaviors of adolescents in Ethiopia [5–9] but there are limited data among HIV positive youth entering adulthood, ages 15–24, where the highest risk of new HIV infections is observed. Further, there are no data on the sexual behaviors regarding the sexual behaviors among those who acquired HIV through perinatal exposure. In the Gondar region of Ethiopia, 25% of the youth had previous sexual intercourse and reported at least one high risk sexual behavior [5]. Knowledge about HIV prevention among young people (15–24) was low 28.4%, and condom use at last higher-risk sex (with a nonmarital, noncohabiting partner) was 65.8% and 28% among males and females respectively [1]. A high number of children are orphaned due to HIV/AIDS in Ethiopia [10] and there were 3% HIV positive children among 15–24 age youth in Ethiopia [11]. We are now seeing perinatally HIV infected children now surviving long enough to join the population of youth and reaching their sexual debut. In this study, we aim to fill the information gap on the sexual behaviors of youth with perinatal and behaviorally acquired HIV infection in northwest Ethiopia.

## 2. Methods

### 2.1. Study Design and Setting

A cross-sectional study among HIV seropositive youths (15–24 years) was conducted in Felege Hiwot referral hospital, Bahrdar, and Debre Markos referral hospital, Debre Markos, Northwest Ethiopia. These hospitals were selected purposively for high client flow and these two big hospitals in west and East Gojjam zones. Felege Hiwot referral hospital is found in the regional city (Bahrdar) which is 555 kilometer from the capital city Addis Ababa and Debre Markos referral hospitals 299 kilometers away from Addis Ababa and 265 kilometers from Bahrdar. The study was undertaken from December 1, 2016, to February 28, 2017.

### 2.2. Study Population

All HIV seropositive youth ages 15–24 years who were receiving medical care and support in the two hospitals were eligible for inclusion in the study. Those youths who were lost to follow up due to drop out transfer out and defaulter status did not enroll in the study.

### 2.3. Sample Size Determination

The sample size was calculated based on the assumption of 95% confidence interval, 5% margin of error, and 50% proportion with high risky sexual practices. The required sample size was calculated using EPI INFO version 3.5, and 384 participants were required to have sufficient power based on the underlying assumptions stated above. However, there were only 350 youths during the study period, and all are included in the study. Study participants were taken during their regular medical follow-up period. Since the data collection period was for three months, almost all youths receiving care at the study sites were enrolled into the study.

### 2.4. Data Collection

Data collection tool was developed from reviewing existing tools in the literature. The questionnaire was translated into Amharic (local language) and back-translated into English to maintain consistency. Ten percent of the tools were pretested in none-selected ART centers and errors identified during the pretest were corrected accordingly. Data were collected by trained interviewers and self-administered questionnaire for illiterate and literate participants respectively. Five case managers (those HIV positive people who had specialized training on HIV case management and working in the hospitals) served as data collectors were recruited and trained by the study team. The training was focused on the objective of the study, confidentiality of information, and the contents of the questionnaire in detail. The primary outcome variable was means of HIV acquisition and youth sexual behavior. For the explanatory variable, sociodemographic characteristics, sexual and other practices that may affect HIV risk, perceived peer-pressure, attending religious ceremony status, the frequency of Religious attending, type of music to which the participant usually listens, knowing another HIV positive person, and HIV/AIDS-related knowledge and attitude were assessed for all youths.

### 2.5. Operational Definitions

Mode of HIV transmission was determined based on respondents answer as (from sexual intercourse, mother to child transmission, using sharp materials (nonoccupational), or I do not know the cause).

Risky sexual practice: inconsistent condom use and or having two or more sexual partners in the last six months were considered as risky sexual behavior [12].

### 2.6. Data Processing and Analysis

Each questionnaire was coded and entered into Epi data version 3.5 statistical package, and it was exported to SPSS version 20.0 statistical package for analysis of statistical inferences. Data cleaning and editing were made before analysis. The result of the study is presented using descriptive statistics (percent, mean, and median values) and analytic statistics (odds ratios).

Before running the multiple logistic regressions assumption of multicolinearity was checked using Pearson correlation and tolerance/variance inflation factor. Binominal logistic regressions were used to calculate the univariate and multivariate-adjusted odds ratio and to determine independent predictors of the dependent variable. Only those variables which were associated with dependent variable with p-value < 0.05 in univariate analysis that were not collinear were used in the multivariate logistic regression model.

### 2.7. Data Quality Management

To maintain data quality training was given for data collectors. Adequately designed data collection material was developed by reviewing different kinds of literature. Supervision is carried out on a daily base to check completeness, consistency both by the supervisor and by investigators. Completed questionnaires were reviewed by the principal investigators. At the completion of data entry, data cleaning was done using frequencies, cross-tabulations, sorting, and listing to check missed values and outliers. Errors that were identified in the data base were corrected by referring to the original paper-based questionnaire. This information in the questionnaires with disagreement with entered data was corrected. But questionnaires with incomplete information were discarded.

### 2.8. Ethical Considerations

The proposal was submitted to Debre Markos University ethics committee for ethical approval and clearance. Permission was also obtained from the hospitals' administrators. For the sake of confidentiality, no personal identifier was recorded in the questionnaire, and data collectors were health professionals working in ART clinic. For children less than 18 years the parents or care givers gave consent. All consents were written informed consent. The participants were informed of the potential risk and benefits of participating in the study and participants were reassured that no personal identifiers would be collected. Those study participants who had symptoms concerning for a sexually transmitted disease (STD) at the time of data collection were referred for clinical care and treatment.

## 3. Result

### 3.1. Socio-Demographic Characteristics

A total of 343 youth were included in this study. More than half 217 (63.3%) were females and mean age of 19.83 (SD of +/- 3.5 years), and 177 (51.6%) were in the age group of 20–24. About 284 (82.8%) were living in urban areas. About 154 (44.9%) receive monthly pocket money. Most of the females, 156 (71.9%), acquired HIV prenatally. (Table 1).

### 3.2. Clinical Characteristics of Youths

The majority of participants 324 (94.5%) were on antiretroviral therapy (ART). About 240 (70%) of them have been told of their HIV status by health professionals in the healthcare facility. Means of HIV acquisition from mother to child was the second most common next to HIV acquired through sexual intercourse (Table 2).

The magnitude of early sexual initiation by age of 15 years is 90 (46.6%) (CI=95% CI (39.2–53.6). Among those with early sexual debut, 94 (90.4%) acquired HIV through perinatal transmission. The majority of youth with perinatal HIV infection reported being sexually active 194 (56.6%) and the average age in years was 17 (SD+/- 3). A larger proportion of the youth reported multiple lifetime sexual partners, 115 (59.3%), with an average of 2 sexual partners. The overall reported HIV status of partners was HIV negative for 50 (63.3%) individuals who had one partner and 67 (58.3%) among those with two or more sexual partners (Table 3).

### 3.3. Factors Associated with Risky Sexual Behavior

Bivariate logistic regressions was run using risky sexual behavior as dependent variable. Independent variables form sociodemographic, clinical, and sexual characteristics. In the bivariate analysis, four variables namely, sex, resident, age and mode of HIV acquisitions were significant. Then in the multivariate logistic regressions revealed that females were 3.09 times more likely to engage in risky sexual practice compared to males AOR, 3.09 (95% CI, 1.41–7.19). Similarly, those acquired HIV through behaviorally were 4.73 times more likely to practice risky sexual behavior AOR, 4.73 (95% CI, 1.32–16.57) Table 4.

## 4. Discussion

This study determines the proportion of youth perinatally acquired HIV in the two large clinical practices and describes the sexual behaviors of these youth. The proportion of youth with perinatally acquired HIV among the 15–24-year olds we studied was high, 35%, minimally lower than the proportion of youth with sexually acquired HIV infection, 38.8%. The high proportion of perinatally acquired infection in this age group is higher than data reported from elsewhere in the world where the majority of HIV acquisition in this age group is related to sexual activity [13–15]. It was estimated that 90% of HIV transmission among children 15 years and younger was from mother to child among HIV infected children [16]. These children are now surviving into their late teens, and as they engage in sexual relations, have the potential to transmit HIV to their HIV-negative sexual partners and, among female youths, perinatal HIV transmission remains possible. This makes effective HIV prevention strategies critically important to address both of these possible modes of transmission, and to break the cycle of ongoing HIV transmission that has contributed to the resurgence of new HIV cases that continue to be reported in our national surveillance data. Our approach to HIV prevention should include a nuanced approach to prevent “transgenerational” HIV infection as this may contribute to increased incidence of new HIV infections that is being reported in many developing countries including Ethiopia. This was indicated where HIV incidence was not decreased as per expected. In 2016, the incidence was similar to what was in 2005 though it was decreased up to 2010 in Ethiopia [1]. Therefore, policymakers better to devise HIV transmission strategy for both sexual and “transgenerational” HIV transmission.

Sexual behavior of HIV-positive youth has not been studied in Ethiopia unlike the general youth in general [6–9]. Further, the differences in sexual behaviors and social characteristics of those who acquired HIV from their mothers has not been previously described. In this study, we describe the contributions of various modes of transmission among HIV-positive youth receiving care.

Majority of females acquired HIV from their mother compared to males. That may explain the higher HIV burden among women among whom HIV prevalence is higher compared with men in Ethiopia [5, 17]. It was also demonstrated that females were more likely to be engaged in risky sexual behavior than males. Majority of youth with perinatally acquired HIV were from rural areas although the majority of HIV-positive youth in the programs are urban residents. This explains the difference in the observed HIV disease burden that is generally higher in urban areas than rural areas. Ninety-one percent of youths in the age group of 20–24 were not perinatally acquired compared to those aged 15–19. With increasing age, there is a higher likelihood of engaging in risky sexual behavior.

Though there is the difference in sex, current residence, monthly pocket money, number of sexual partners, age, and living arrangement, there was no difference by early initiation of sexual intercourse, consistent condom use and ever use a condom comparing youth with perinatally acquired HIV infection and youth with sexual and other modes of HIV acquisition. This study did not find a causal relationship between mode of HIV acquisition and other youth characteristics.

## 5. Conclusion

More children who acquired the virus from their mother are joining the youth population than ever. Most of their sexual behavior is similar to those who acquired HIV through sexual and sharp material and those who did know the source. Youths above 20 years and those with multiple sexual partners who acquired HIV were less likely to have acquired HIV through perinatal HIV transmission. Youths in urban setting and living with relatives were less likely to receive the virus from their mother.

## Figures and Tables

**Table 1:** sociodemographic characteristics of HIV positive youths by HIV acquisition status in North West Ethiopia, 2017.

Characteristics	HIV acquisition through (%)		Total (%)	P-value
	Mother to child	Behavioral		
**Sex **				
Male	67 (53.2)	59 (46.8)	126 (36.7)	0.001
Female	156 (71.9)	61 (28.1)	217 (63.3)	
**Age**				
15–19	54 (32.5)	112 (67.5)	166 (48.4)	0.001
20–24	169 (95.5)	8 (4.5)	177 (51.6)	
**Current residence**				
Rural	51 (32.5)	8 (13.6)	59 (17.2)	0.001
Urban	172 (60.6)	112 (39.4)	284 (82.8)	
**Marital status**				
Married/cohabited	97 (90.7)	10 (9.3)	107 (31.2)	0.001
Single	78 (42.4)	106 (57.6)	184 (53.6)	0.001
Divorced	38 (95)	2 (5)	40 (11.7)	0.001
Widowed	10 (83.3)	2 (16.7)	12 (3.5)	0.093
**Have monthly income**				
Yes	131 (84.5)	24 (15.5)	155 (44.9)	0.001
No	92 (48.9)	96 (51.1)	188 (55.1)	
**Amounts of monthly income**				
50–700 birr	64 (78)	18 (22)	82 (53.2)	0.016
>700 birr	66 (91.7)	6 (8.3)	72 (46.8)	
**Currently living with**				
Nuclear family	52 (40.3)	77 (59.7)	129 (37.6)	0.001
Relatives	23 (42.6)	31 (57.4)	54 (15.7)	0.001
Alone	84 (9.3)	9 (9.7)	93 (27.1)	0.001
Husband/wife	64 (95.5)	3 (4.5)	67 (19.5)	0.001
**Parents residence**				
Urban	109 (51.4)	103 (48.6)	212 (61.8)	0.001
Rural	114 (87)	17 (3)	131 (38.2)	
**Education status**				
Cannot read and Write	45 (97.8)	1 (2.2)	46 (13.4)	0.001
Can read and write only	36 (94.7)	2 (5.3)	38 (11.1)	0.001
Complete primary education	80 (51.3)	76 (48.7)	156 (45.5)	0.001
Complete secondary education	62 (60.2)	41 (39.8)	103 (30)	0.001

**Table 2:** Sociodemographic characteristics among HIV positive youths in North West Ethiopia, 2017.

Characteristics	Response (frequency and percentage)
**ART status**	
On ART	324 (94.5)
Pre ART	19 (5.5)
**Who tolled their HIV status**	
Parents	81 (23.6)
Health professionals	240 (70)
Their friends	22 (6.4)
**HIV acquired through**	
Sexual intercourse	133 (38.8)
Mother to child	120 (35)
Sharp materials	27 (7.9)
did not know	63 (18.4)

**Table 3:** Self-reported sexual behavior by HIV acquisition status among HIV positive youths in North West Ethiopia, 2017.

**Characteristics**	**HIV acquisition status**		**Total (%)**
	**Mother to child**	**Others means**	
**Had sex in the last six months**			
Yes	30 (83.33%)	6 (16.67%)	36 (10.50)
No	193 (62.87%)	114 (37.13%)	307 (89.5)
**Ever had sex**			
Yes	172 (88.7%	22 (11.3%)	194 (56.6)
No	51 (34.2%)	98 (65.8%)	149 (43.4)
**First sexual initiation started**			
Less than 18 years	94 (90.4%)	10 (9.6%)	90 (46.4)
Greater than or equal to 18 years	78 (86.7%	12 (13.3%)	104 (53.6)
**Numbers of lifetime sexual partner**			
single	63 (79.7%)	16 (11.3%)	79 (40.7)
multiple	110 (95.7)	15 (4.3%)	115 (59.3)
**HIV status of sexual partner (have only 1)**			
HIV positive	14 (82.4%)	3 (17.6%)	17 (21.5)
HIV negative	40 (80%)	10 (20%)	50 (63.3)
Unknown	12 (100%)	0	12 (15.2)
**HIV status of sexual partner (more than 1)**			
HIV positive	19 (90.5%)	2 (9.5)	21 (18.3)
HIV negative	63 (94%)	4 (6%)	67 (58.3)
Unknown	23 (88.9%	3 (11.1%)	27 (23.5)
**Ever use condom**			
Yes	119 (86.9%)	18 (13.1%)	137 (70.6)
No	54 (94.4%)	3 (5.3%)	57 (29.4)
**Consistent condom use**			
Yes	65 (84.4%)	12 (15.6%)	77 (56.2)
No	54 (90%)	6 (10%)	60 (43.8)

**Table 4:** Univariate and multivariate logistic regression of risky sexual behaviors of HIV positive youths in northwest Ethiopia, 2017.

**Characteristics**	**Risky sexual behavior**		**Crud odds ratio at 95% CI**	**Adjusted odds ratio at 95% CI**
	Yes	No		
**Sex**				
Male	35	14	1	1
Female	79	66	2.09 (1.04–4.21)	**3.18 (1.41**–**7.19)**
**Residence**				
Rural	33	11	1	1
Urban	81	69	2.56 (1.20–4.43)	2.14 (0.97–4.69)
**Age**				
15–19	12	23	1	1
20–24	102	57	0.29 (0.14–0.63)	0.49 (0.20–1.18)
**Mode of HIV acquisitions**				
MTCT	109	64	1	1
behavioral	5	16	5.45 (1.91–15.58)	**4.73 (1.35**–**16.57)**

## Data Availability

The datasets generated and analyzed during the current study are available and can be accessed from the corresponding author via collaboration request.
